# Association of *MC4R* (rs17782313) with diabetes and cardiovascular disease in Korean men and women

**DOI:** 10.1186/s12881-020-01100-3

**Published:** 2020-08-17

**Authors:** Jae Woong Sull, Gitae Kim, Sun Ha Jee

**Affiliations:** 1 Department of Biomedical Laboratory Science, College of Health Sciences, Eulji University, Seongnam, Korea; 2 Department of Epidemiology and Health Promotion, Institute for Health Promotion, Graduate School of Public Health, Yonsei University, Seoul, Korea

**Keywords:** Fasting glucose, *MC4R*, Polymorphisms

## Abstract

**Background:**

Diabetes is mostly assessed by the fasting glucose level. Several studies reported that serum fasting glucose levels and cardiovascular disease are associated with MC4R.

**Methods:**

A total of 4294 subjects participated in this study. There were 1810 subjects with cardiovascular disease among the 4294 subjects. We used multivariate linear regression models and multiple logistic regression analysis.

**Results:**

Individuals with the TC/CC genotype had a 1.29-fold higher risk of diabetes than did those with the TT genotype when adjusting for age, sex, and BMI (OR, 1.29; 95% CI, 1.04–1.60). For healthy subjects, the association was significant in women (OR, 1.99; 95% CI, 1.01–3.93). Men with the TC/CC genotype had a 1.21-fold higher risk of cardiovascular disease than did those with the TT genotype when adjusting for age, sex, and BMI (OR, 1.21; 95% CI, 1.04–1.41). The relationship between *MC4R* and cardiovascular disease was stronger in lean men (OR, 1.40; 95% CI, 1.12–1.74, *p* = 0.0028) than in overweight men.

**Conclusions:**

This study suggests that the rs17782313 SNP in *MC4R* is related to diabetes and the SNP is also associated with cardiovascular disease in lean men.

## Background

Diabetes is mainly assessed by the fasting blood-glucose level [1]. Several previous studies reported that the melanocortin-4 receptor (*MC4R*) (MIM 155541) gene is a candidate as a causal gene for type 2 diabetes [2, 3]. *MC4R* deficiency is related to monogenic obesity [4]. The *MC4R* rs17782313 SNP has also been linked with obesity in Europeans and Koreans [5, 6]. The SNP rs17782313 was also associated with diabetes in several studies [3, 7, 8]. In Korea, obesity increases the risk of death from cardiovascular disease [9].

Recent studies have reported that the *MC4R* gene was related to cardiovascular disease [10, 11]. In this study, we investigated the relationship between diabetes, cardiovascular disease, and the rs17782313 *MC4R* SNP in Korean men and women. We also evaluated modification of the relationship of *MC4R* and cardiovascular disease by obesity.

## Methods

### Study population

A total of 4294 subjects were the participants who had general health examinations in Health Promotion Center in University hospitals [12]. The biological samples for DNA extraction were obtained prospectively between 2004 and 2013 as a part of the Korean Cancer Prevention Study-II (KCPS-II) Biobank [13]. There were 1810 subjects with Cardiovascular Disease (CVD) among the 4294 subjects. The cases were obtained by the NHIC health-insurance reimbursement data. The codes of the International classification of Disease (ICD), 10th Revision (I00-I99), were used for the definition of CVD. Because of missing fasting blood-glucose level, body mass index (BMI), and SNP rs17782313 data, 35 subjects were excluded. Therefore, the final subjects included 4259 people, and 1782 subjects among them were Cardiovascular Disease (CVD) patients. Among 1782 Cardiovascular Disease (CVD) patients, 1409 individuals had ischemic heart disease and 868 had stroke. The other 2477 subjects were the healthy subjects. The healthy subjects were defined as subjects who do not have the cardiovascular disease.

### Data collection

Each participant was interviewed using a structured questionnaire to collect their personal history of smoking status (never smoked, ex-smoker or current smoker) and demographic characteristics (age, sex, etc.), and other characteristics such as medical history and any medications. Participants were defined as ‘current smokers’ if they were smoking currently, ‘never smokers’ if they had no prior history of smoking and ‘ex-smokers’ if they had previously smoked but at the time of measurement did not smoke. The weight and height were measured with participants lightly clothed.

Peripheral venous-blood samples taken after a 12-h fast and stored at − 70 °C were used for the measure of fasting blood sugar (FBS), total cholesterol, triglycerides, and HDL-C. We used a Hitachi-7600 analyzer (Hitachi, Ltd., Tokyo, Japan) for the clinical chemistry assays. Detailed phenotype data were previously described [12, 13].

### Genotyping assays

The TaqMan reaction was used for the genotyping of the rs17782313 MC4R gene SNP [14]. Only the SNPs with a concordance rate > 99% in duplicates and a genotype success rate > 98% were included.

### Statistical analysis

Data are shown as means ± standard deviation. PLINK and SAS ver. 9.4 (SAS Institute, Cary, NC, USA) were used for most statistical analyses. The linear regression under the additive genetic model was used to assess the association of MC4R rs17782313 with fasting blood glucose levels considering age and sex as covariates. We also used multiple logistic regression analysis under the recessive model to examine the association of the MC4R rs17782313 with diabetes and cardiovascular disease. Body mass index was divided by the median values. The association between the MC4R SNP, diabetes, and cardiovascular diseases were expressed by Odds ratios (ORs) with 95% confidence intervals (CIs). Diabetes was defined as fasting serum glucose ≥126 mg/dL or medication. All statistical tests were two-sided, and *p* < 0.05 was used for the statistical significance.

## Results

The mean age in men and in women was 51.9 and 52.7 (Table 1). Diabetes patients were about 8.9% of the subjects; 37.3% in men and 3.9% in women were current smokers of the sample dataset. Cardiovascular disease patients were 44.7% of men and 35.9% of women. Table 2 shows the *p* values from a linear regression model for FBS levels when age and sex were included as covariates. The rs17782313 SNP in the *MC4R* gene was related to mean FBS level and BMI (effect per allele, 1.542 mg/dL, *p* = 0.0057, and 0.227 mg/dL, *p* = 0.0018). For healthy individuals, the rs17782313 SNP in the *MC4R* gene was related to mean FBS level and BMI (effect per allele, 1.477 mg/dL, *p* = 0.0205, and 0.237 mg/dL, *p* = 0.0096).

**Table 1:** General characteristics of the study population

Subjects		All	Men	Women
N		4259	2896	1363
		Mean ± SD	Mean ± SD	Mean ± SD
Age, year		52.2 ± 10.2	51.9 ± 10.3	52.7 ± 10.1
Weight, kg		67.4 ± 11.1	71.8 ± 9.6	58.1 ± 7.7
Body mass index, kg/m^2^		24.4 ± 3.0	24.8 ± 2.7	23.5 ± 3.2
Fasting blood sugar, mg/Dl		97.0 ± 22.6	98.8 ± 24.0	93.1 ± 18.7
Systolic blood pressure, mmHg		121.9 ± 14.5	123.3 ± 13.9	118.9 ± 15.3
Diastolic blood pressure, mmHg		78.2 ± 10.8	79.7 ± 10.6	75.0 ± 10.5
		%	%	%
Smoking status	Ex	28.4	40.1	2.4
	Current	26.9	37.3	3.9
Cardiovascular disease		41.8	44.7	35.9
Diabetes^a^		8.9	10.2	6.2
Family history of diabetes		14.5	14.1	15.4

*SD* standard deviation

^a^ Diabetes was defined as fasting serum glucose ≥126 mg/dL or medication

**Table 2:** Association between the rs17782313 single nucleotide polymorphism in the MC4R gene and fasting blood-sugar levels based on a linear regression model

	Genotypes				
Phenotypes	TT	TC	CC	Effect (mg/dL)	*P*-value
	Mean ± SD	Mean ± SD	Mean ± SD		
All subjects	(*N* = 2428)	(*N* = 1578)	(*N* = 253)		
Fasting blood sugar, mg/dL	96.0 ± 21.3	98.6 ± 24.9	96.5 ± 18.4	1.542	0.0057*
Body mass index, kg/m^2^	24.3 ± 2.9	24.5 ± 3.0	24.8 ± 3.2	0.227	0.0018*
Weight, kg	67.2 ± 11.0	67.6 ± 11.1	68.6 ± 11.1	0.739	0.0010*
Systolic blood pressure, mmHg	121.7 ± 14.6	122.0 ± 14.3	122.7 ± 15.0	0.452	0.2032
Diastolic blood pressure, mmHg	78.2 ± 11.0	78.2 ± 10.4	78.4 ± 11.2	0.061	0.8184
	%	%	%		
Cardiovascular disease	40.7	44.1	38.3		0.0543
Diabetes	8.0	10.5	7.1		0.0153
Healthy subjects	(*N* = 1439)	(*N* = 882)	(N = 156)		
Fasting blood sugar, mg/dL	93.6 ± 18.5	96.3 ± 22.5	93.7 ± 13.3	1.477	0.0205*
Body mass index, kg/m^2^	24.0 ± 2.9	24.2 ± 3.0	24.3 ± 2.6	0.237	0.0096*
Weight, kg	66.5 ± 11.2	66.9 ± 11.5	67.8 ± 10.6	0.839	0.0035*
Systolic blood pressure, mmHg	119.4 ± 13.5	119.3 ± 13.3	119.8 ± 12.1	0.156	0.7119
Diastolic blood pressure, mmHg	78.8 ± 10.9	78.5 ± 10.5	78.8 ± 10.1	−0.039	0.9068
	%	%	%		
Diabetes	5.7	8.1	5.1		0.0642

Estimated effect size (β) and *p* values in the multiple linear regression model considered age and sex in the additive model. *P* values for cardiovascular disease and diabetes were obtained from chi-square test. * significant of *p* < 0.05

The relationship between diabetes and the *MC4R* gene SNP rs17782313 was examined (Table 3). Individuals with the TC/CC genotype had a 1.29-fold higher risk of diabetes than did those with the TT genotype when adjusting for age, sex, and BMI (OR, 1.29; 95% CI, 1.04–1.60). When analyzed by sex, the relationship between *MC4R* and diabetes was significant only for men (OR, 1.33; 95% CI, 1.04–1.70), not women (OR, 1.10; 95% CI, 0.70–1.75). For healthy subjects, individuals with the TC/CC genotype had a 1.40-fold higher risk of diabetes than did those with the TT genotype when for adjusting age, sex, and BMI (OR, 1.40; 95% CI, 1.01–1.95). However, the association was stronger in women (OR, 1.99; 95% CI, 1.01–3.93), and the association of MC4R with diabetes was not found in men (OR, 1.24; 95% CI, 0.85–1.81).

**Table 3:** Odds ratios (OR) of the polymorphic rs17782313 MC4R genotypes for diabetes in the population

		Normal	Diabetes ^b^				
				Model 1		Model 2	
Subjects	Genotype	*N* (%)	*N* (%)	OR (95% CI^a^)	*P*-value	OR (95% CI^b^)	*P*-value
All	TT	2233 (57.6)	195 (51.5)	1.00 (reference)		1.00 (reference)	
(*n* = 4259)	TC /CC	1647 (42.4)	184 (48.6)	1.32 (1.07–1.64)*	0.0113	1.29 (1.04–1.60) *	0.0218
Men	TT	1513 (58.2)	151 (51.4)	1.00 (reference)		1.00 (reference)	
	TC/ CC	1089 (41.8)	143 (48.6)	1.34 (1.05–1.72) *	0.0182	1.33 (1.04–1.70) *	0.0219
Women	TT	720 (56.3)	44 (51.8)	1.00 (reference)		1.00 (reference)	
	TC/ CC	558 (43.7)	41 (48.2)	1.25 (0.80–1.96)	0.3337	1.10 (0.70–1.75)	0.6878
All Healthy	TT	1357 (58.6)	82 (50.9)	1.00 (reference)		1.00 (reference)	
(*n* = 2477)	TC /CC	959 (41.4)	79 (49.1)	1.46 (1.05–2.02) *	0.0234	1.40 (1.01–1.95) *	0.0424
Men	TT	887 (59.9)	67 (54.9)	1.00 (reference)		1.00 (reference)	
	TC/ CC	594 (40.1)	55 (45.1)	1.27 (0.88–1.85)	0.2082	1.24 (0.85–1.81)	0.2580
Women	TT	470 (56.3)	15 (38.5)	1.00 (reference)		1.00 (reference)	
	TC/ CC	365 (43.7)	24 (61.5)	2.13 (1.09–4.19) *	0.0277	1.99 (1.01–3.93) *	0.0490

Model 1: Adjusted for age and sex. Model 2: Adjusted for age, sex, and body mass index

^a^ CI, confidence interval. ^b^ Diabetes was defined as fasting serum glucose ≥126 mg/dL or medication. * significant of *p* < 0.05

The relationship of the *MC4R* gene SNP rs17782313 to cardiovascular disease was also examined (Table 4). Men with the TC/CC genotype had a 1.21-fold (range, 1.04–1.41-fold) higher risk of cardiovascular disease than did those with the TT genotype when for adjusting age, sex, BMI (OR, 1.21; 95% CI, 1.04–1.41). In contrast, the relationship of MC4R with cardiovascular disease was not found in women.

**Table 4:** Odds ratios (OR) of the polymorphic rs17782313 MC4R genotypes for cardiovascular disease in the population (*n* = 4259)

Subjects		Normal	Cardiovascular Disease				
				Model 1		Model 2	
	Genotype	*N* (%)	*N* (%)	OR (95% CI^a^)	*P*-value	OR (95% CI)	*P*-value
All	TT	1439 (58.1)	989 (55.5)	1.00 (reference)		1.00 (reference)	
	TC /CC	1038 (41.9)	793 (44.5)	1.13 (1.00–1.28)	0.0599	1.11 (0.98–1.26)	0.1174
Men	TT	954 (59.5)	710 (54.9)	1.00 (reference)		1.00 (reference)	
	TC/ CC	649 (40.5)	583 (45.1)	1.22 (1.05–1.42)*	0.0096	1.21 (1.04–1.41)*	0.0144
Women	TT	485 (55.5)	279 (57.1)	1.00 (reference)		1.00 (reference)	
	TC/ CC	389 (44.5)	210 (42.9)	0.94 (0.75–1.19)	0.5990	0.89 (0.71–1.13)	0.3474

Model 1: Adjusted for age and sex

Model 2: Adjusted for age, sex, and body mass index

* significant of *p* < 0.05

^a^ CI, confidence interval

Table 5 shows the analysis by BMI for men. The relationship between *MC4R* and cardiovascular disease was stronger in men with BMI < 24.75 (OR, 1.40; 95% CI, 1.12–1.74, *p* = 0.0028) than in subjects with BMI > = 24.75 (*p* = 0.6461). However, the interaction between BMI and *MC4R* (rs17782313) genotype for cardiovascular disease was not significant (*p* for interaction = 0.0753).

**Table 5:** Odds ratios (OR) of polymorphic rs17782313 MC4R genotypes for cardiovascular disease in Korean men (*n* = 2896)

		Normal	Cardiovascular Disease		
Subjects	Genotype	*N* (%)	*N* (%)	OR^a^ (95% CI^b^)	*P*-value
BMI < 24.75	TT	516 (61.4)	325 (53.4)	1.00 (reference)	
	TC/ CC	325 (38.6)	284 (46.6)	1.40 (1.12–1.74)*	0.0028
BMI > =24.75	TT	438 (57.5)	385 (56.3)	1.00 (reference)	
	TC/ CC	324 (42.5)	299 (43.7)	1.05 (0.85–1.30)	0.6461

* significant of *p* < 0.05

*P* for the interaction between obesity (BMI > = 24.75) and MC4R (rs17782313) =0.0753

^a^ Adjusted for age and BMI ^b^ CI, confidence interval

## Discussion

In a cohort of 4259 people, the rs17782313 SNP in the *MC4R* gene was related to serum glucose level, as in previous studies. A meta-analysis of 123,373 individuals reported that the rs17782313 polymorphism in MC4R gene has the BMI independent significant association with risk of type 2 diabetes [7]. A recent study reported a strong association with MC4R loci for type 2 diabetes (OR = 1.70) [2]. Another study reported that the rs17782313 SNP was significantly associated with increased risk of diabetes [3]. A recent study reported that a Mediterranean dietary pattern could influence the relationship between MC4R gene rs17782313 polymorphisms and the risk of type 2 diabetes [8]. In our study for healthy subjects, the association of MC4R gene rs17782313 with diabetes was stronger in women than in men. A study also reported that the allele C of MC4R (rs17782313) was associated with a higher risk of type 2 diabetes mellitus in women [15].

The MC4R gene was associated with BMI and is involved in the regulation of insulin secretion [5, 16]. Loos et al. [5] in a meta-analysis from European subjects reported a significant association between rs17782313 in the MC4R gene and BMI in adults and children. A study also demonstrated that genetic variants in MC4R affect the obesity phenotype in Koreans [6]. In another meta-analysis, the association of the MC4R gene with insulin resistance and type 2 diabetes was reported even after adjustment for BMI [7]. The MC4R gene (rs17782313 and rs17700633) were related to obesity risk and insulin resistance in two genome-wide association studies [5, 17]. In this study, we found that the MC4R SNP rs17782313 was associated with BMI and weight.

In this study, the rs17782313 SNP in the MC4R gene was related to cardiovascular disease in men. The association was stronger in lean men than in overweight men. A recent study reported that MC4R gene may contribute the co-occurrence of coronary artery disease and obesity [10]. They reported that the MC4R gene SNPs were associated with coronary artery disease (*p* < 5 × 10^− 8^). They also explained that the mechanisms whereby MC4R SNPs contribute to obesity can increase the liability to coronary artery disease. Another study reported that the MC4R showed higher expression in mesenteric fat among obese and diabetic rats then compared with lean rats [18]. In the present study, the mean age in men with BMI < 24.75 (52.6 years old) was higher than in men with BMI > = 24.75 (51.3 years old) (*p* = 0.0012). The CVD patients in men with BMI < 24.75 (42.0%) was smaller than in men with BMI > = 24.75 (47.3%) (*p* = 0.0046) (Data not shown).

MC4R is localized to chromosome 18q21.3 [19], is highly expressed in the hypothalamus, and is related to appetite and energy control [20]. In mice research, the MC4R gene is associated with hyperinsulinemia before the onset of extreme obesity [21]. Lipocalin-2 crosses the blood brain barrier and binds to MC4R in the paraventricular and ventromedial neurons of the hypothalamus. A recent loss- and gain-of-junction experiment in mice reported that osteoblast-derived lipocalin-2 maintains glucose homeostasis [22]. Another recent study reported that the MC4R gene SNPs influence the body fat content and distribution, as well as relative increase in postprandial carbohydrate utilization [23].

This study has some limitations that need to be discussed. There were the differences in the baseline characteristics including smoking rate, cardiovascular disease, and diabetes prevalence by gender. When we assessed the distribution of the MC4R alleles by gender, the genotype frequency of MC4R gene was similar between men and women (Data not shown). Another limitation is that this study subjects included many CVD patients because the CVD cases were obtained from the KCPS2 dataset (*n* = 156,701) for the case control study design [13]. Therefore, we analyzed the association between diabetes and MC4R gene in the healthy subjects who did not have the cardiovascular disease.

## Conclusion

Asian people may have genetic backgrounds different from those of Western individuals [24]. However, this Korean cohort showed that the rs17782313 SNP in the MC4R gene is related to diabetes and obesity. The SNP was also associated with cardiovascular disease in lean men.

## Data Availability

The datasets generated during and/or analyzed during the current study are not publicly available due to data security reasons, i.e., the data contain potentially participant identifying information, but are available from the corresponding author on reasonable request.

## Abbreviations

CI: Confidence intervals
CVD: Cardiovascular disease
FBS: Fasting blood sugar
MC4R: Melanocortin-4 receptor
BMI: Body mass index
ICD: International classification of Disease
OR: Odds ratios
SD: Standard deviation
SNP: Single nucleotide polymorphism
